# Names in Nursing: A Call for Definitions in Terms

**Published:** 1916-07-29

**Authors:** 


### THE MATRONS' AND SISTERS' DEPARTMENT.

### NAMES IN NURSING.

### The Call for Definitions in Terms.

In the present day throughout the nursing profession there is an increasing habit to use initial letters when writing or speaking, which since the war has been so extended as to make the conversation of even representative members of the profession as difficult to follow as the universal language Esperanto. Thus a soldier described as an "I. H." was found to be invalided home. This habit has its humorous side, for, as in the case of scientists where the terminology is so overdone as to confound the professor in every branch but his own, nurses at home have frequently to stop a speaker from the Front, and *vice versa*, to secure an explanation of the mysterious letters so fluently uttered and so ever-increasing. The habit has its evil side, too, for to it we have traced the abuse of the nurse's uniform by the unqualified and the heathen, who, recognising the attractiveness of the fabrics employed, which embrace all colours, use the privilege of their sex to dress themselves in the particular shade which suits their own complexion best. Hence garrulity and the all-embracing taste in fabrics and colours may present many pitfalls to the average nurse. It follows that everybody will welcome registration, because it has become known that the agreed Bill will cure the uniform evils by obtaining statutory powers for every registered nurse to wear a defined badge or uniform which no other person may use without liability, on summary conviction, to a heavy fine. The habit of initial letters in lieu of words must eventuate in boredom and so terminate in its suicide.

But seriously there is a distinct call for accepted definitions of the terms used in nursing. One swallow does not make a summer, but one good incident, resting upon high legal utterance, may suffice to enforce this point. The College of Nursing and the registration of certificated trained nurses are both well on their way to fruition. They mean higher standards upheld by discipline resting upon statutory powers enforceable in the Law Courts. Every woman in exact proportion to her competence, high training, and education will speedily find her proper place in the nursing profession. Clearness of definition in regard to titles will thus become of the first importance, a fact every nurse who is proud of her profession should learn to recognise and enforce. Parliament cannot grant protection to slang words or manifold titles. All it can do is to place it in the power of fully-trained members of the nursing profession to possess the right to the sole use of the term registered nurse, registered male nurse, or registered mental nurse.

Recent cases in the Law Courts have shown that judges of the High Court, and County Court judges too, especially when they are well disposed to nurses, are often at sea when they have before them cases where one of the parties is described as a nurse, though she may not have been properly trained or possess any recognised claim to use the term. The *Nursing Mirror* of the 22nd instant reports a case in point, where the Master of the Rolls in Dublin upheld the employment on a brass plate of the words "Certified Ladies' Nurse," and expressed dissent from the suggestion "that the profession of ladies' nurse detracted from the social position of the person using it." He even declared that he considered it a most honourable profession. In reply to a request to his lordship to explain his meaning, the Right Hon. Charles O'Connor, K.C., Master of the Rolls in Ireland, properly insists that he ought not to express "publicly and extra-judicially any opinion on a question which seems to be the subject of legal controversy." His lordship further proceeds: "I have no hesitation in affirming that the profession of ladies' nurse is a highly honourable one which, so far from detracting from, ought to exalt the social position of any woman who adopts it." His lordship has no experience of the ladies' nursing profession outside of Ireland. It would be instructive to hear how many people there are in Great Britain who have any experience of the ladies' nursing profession within its area. Mr. O'Connor, in the course of his letter, makes it clear that the nurse he refers to is a fully-trained certificated nurse, though his words would seem to convey that the term ladies' nurse relates to those following a distinct branch of nursing, which appears not to be the case. Again, and still more remarkable, is the utterance of Mr. Justice Bargrave Deane, who in a recent case referred to in THE HOSPITAL (March 11, 1916, page 533) remarked: "But this lady was not even a nurse; she was a nursing sister." Yet the judge ultimately decided that a letter written by the deceased lady in question should be regarded as a "soldier's will," and enforced accordingly.

These cases afford ground for action on the part of the College of Nursing, the Council of which will no doubt consider the desirability of preparing and issuing a code of nursing axioms including definitions of the various grades or ranks in nursing—*i.e.*, probationer, nurse, sister, matron, lady superintendent, and the infinite number of titles of relatively recent creation which apply to the offices held by trained nurses in connection with municipal, health, school, private, and other sections of the nursing field. It must be clear that the approaching legislation conferring statutory powers and definite titles, with the highest standards of competency, will entail an early issue of precise axioms and definitions in some such form as that suggested, for the information and guidance not only of the judge in his Court, but of all sections of the community.

